# The landscape of genetic alterations in ameloblastomas relates to clinical features

**DOI:** 10.1007/s00428-018-2305-5

**Published:** 2018-02-01

**Authors:** Sibel Elif Gültekin, Reem Aziz, Carina Heydt, Burcu Sengüven, Joachim Zöller, Ali Farid Safi, Matthias Kreppel, Reinhard Buettner

**Affiliations:** 1 Department of Oral Pathology, Faculty of Dentistry, Gazi University, Ankara, Turkey; 2 Cologne Institute of Pathology, University Hospital Cologne, Kerpener Straße 62, 50937 Cologne, Germany; 3 Clinic for Oral and Maxillofacial Surgery, University Hospital Cologne, Cologne, Germany

**Keywords:** Ameloblastoma, MAPKinase signaling, Hedgehog signaling, Mutational risk profiling, Genotype-phenotype correlation, Mutation-based risk stratification

## Abstract

Ameloblastoma is a mostly benign, but locally invasive odontogenic tumor eliciting frequent relapses and significant morbidity. Recently, mutually exclusive mutations in *BRAF* and *SMO* were identified causing constitutive activation of MAPK and hedgehog signaling pathways. To explore further such clinically relevant genotype-phenotype correlations, we here comprehensively analyzed a large series of ameloblastomas (98 paraffin block of 76 patients) with respect to genomic alterations, clinical presentation, and histological features collected from the archives of three different pathology centers in France, Germany, and Turkey. In good agreement with previously published data, we observed *BRAF* mutations almost exclusively in mandibular tumors, *SMO* mutations predominantly in maxillary tumors, and single mutations in EGFR, KRAS, and NRAS. KRAS, NRAS, PIK3CA, PTEN, CDKN2A, FGFR, and CTNNB1 mutations co-occurred in the background of either *BRAF* or *SMO* mutations. Strikingly, multiple mutations were exclusively observed in European patients, in solid ameloblastomas and were associated with a very high risk for recurrence. In contrast, tumors with a single *BRAF* mutation revealed a lower risk for relapse. We here establish a comprehensive landscape of mutations in the MAPK and hedgehog signaling pathways relating to clinical features of ameloblastoma. Our data suggest that ameloblastomas harboring single *BRAF* mutations are excellent candidates for neo-adjuvant therapies with combined BRAF/MEK inhibitors and that the risk of recurrence maybe stratified based on the mutational spectrum.

## Introduction

Ameloblastoma is a mostly benign, locally invasive odontogenic neoplasm arising in the jaws. The tumor originates from the epithelium involved in tooth formation, the enamel organ, epithelial cell rests of Malassez, reduced enamel epithelium, and epithelial lining of odontogenic cysts with special reference to dentigerous cysts [1, 2].

Tooth development (odontogenesis) is being initiated by interactions between epithelial and mesenchymal cells derived from the ectoderm of the first branchial arch and the ectomesenchyme of the neural crest. Odontogenesis involves several morphologically distinct stages. The mesenchyme of the developing tooth induces epithelial proliferation forming the dental lamina at the sixth week of gestation. Small epithelial cell nests invade the underlying mesenchyme and are referred to as bud stage. This phase is followed by proliferation and condensation of the mesenchyme around the bud, which then invaginates to form a cap shape known as cap stage at gestational weeks 9 to 11. The enamel organ starts being formed in the cap stage, then further differentiates into an outer and inner enamel epithelium, and further forms the stellate reticulum, imparting a bell shape, known as the bell stage. Ameloblasts arise from the inner epithelium. Ameloblastic epithelium shows nuclear polarization toward the underlying reticulum surface, known as the stratum intermedium thought to assist enamel production, which is not present in ameloblastomas, and therefore, no enamel is being produced. Epithelial polarization is recapitulated in ameloblastic tumors and marks the formation of pre-ameloblasts, which together with odontoblasts from the dental papilla induce dentin and enamel [3].

Ameloblastomas represent approximately 1% of all oral tumors and about 9 to 11% of all odontogenic tumors. Approximately 80% of ameloblastomas arise in the mandible, foremost in the third molar region, and the remaining 20% in the maxilla [1, 2]. Ameloblastomas were classified by the World Health Organization (WHO) into solid/multicystic, peripheral (extraosseous counterpart of the intraosseous solid/multicystic ameloblastoma), desmoplastic, and unicystic types with implications for treatment [4]. However, in the new 2017 classification of WHO, ameloblastomas were narrowed to ameloblastoma (conventional), unicystic, extraosseous/peripheral, and metastasizing variants due to the introduction of prospective views based on updates from genetic studies [4].

Ameloblastomas typically present as locally aggressive odontogenic tumors, frequently asymptomatic and slow-growing, with no evidence of swelling. There is a high propensity for local recurrence if not adequately removed at the initial surgery and, though the tumor may appear microscopically benign, development of distant metastasis is possible. Ameloblastic tumors with cytological atypia are classified as ameloblastic carcinoma and have a propensity for rapid growth and metastasis. Rarely, ameloblastic neoplasms metastasize despite of a benign histologic appearance, and these tumors are classified as metastasizing ameloblastomas [1, 2]. Current treatment options for ameloblastomas include both conservative treatment (enucleation or curettage) and resection. The former is associated with high rates of recurrence, while the latter results in significant facial deformity and morbidity. Importantly, patients should be followed up life-long due to an unpredictable biological behavior, especially in the case of maxillary tumors which carry a worse overall prognosis as compared to mandibular ameloblastomas [1, 4].

Development of non-invasive therapies has been precluded by a lack of understanding the molecular pathology of ameloblastomas, and hence, no risk classification for the likelihood of recurrence is currently available. Low prevalence, frequently small tissue samples and degradation of DNA resulting from decalcification has not made ameloblastoma an easy candidate for molecular analyses. However, comprehensive and highly sensitive next-generation sequencing technologies paved the way. Recently, oncogenic mutations were discovered activating constitutively signal transduction pathways relating to developmental stages of odontogenesis including the mitogen-activated protein kinase (MAPK) and hedgehog pathways [5–11].

These studies identified *BRAF* as the most frequently mutated gene causing constitutive activation of the MAPK pathway in mandibular ameloblastomas of younger age patients, whereas *SMO* mutations were identified to activate hedgehog signaling predominantly in maxillary ameloblastomas of older patients [6–8]. Since in very low frequencies the two mutations coexist, it has been proposed that these genetic alterations define two etiologically independent ameloblastic entities [5, 8]. However, the emerging genetic dichotomy between the tumors of two anatomic locations and age with regard to *BRAF* and *SMO* is not yet fully elucidated. Moreover, it is clinically important to correlate mutational status and histological evaluation with clinical outcome in large multicenter case series [5]. To explore further such clinically relevant genotype-phenotype features, we here comprehensively analyzed a large series of ameloblastomas collected from the archives of three different centers from Germany, France, and Turkey.

## Material and methods

### Tumor specimens, histological, clinical, and radiological data

Our study included a total of 98 paraffin blocks of 76 patients presenting with ameloblastomas between the years 2001 and 2015 collected from the archives of the Institute of Pathology, University Hospital Cologne/Germany (*n* = 26), Department of Pathology, University Hospital Rouen/France (*n* = 9), and from the Department of Oral Pathology, Dental Faculty, Gazi University Ankara/Turkey (*n* = 41). Experimental protocols were reviewed and approved by the Ethics Committee of the University of Cologne (no. 13-091), University Hosital Rouen (DC 2008_689), and Gazi University (no. 77082166-604.01.02-2017-03).

Clinical and histologic characteristics of all cases are summarized in Tables 1 and 2. The mean age at diagnosis was 48.4 years (range 9 to 92 years). Forty-eight patients were males and 28 females. Tumors were distributed throughout the jaws with 55 cases in mandibular, 21 cases in maxilla, 28 cases in right-sided, 30 cases in left-sided, and 13 cases in an anterior location. No clinical data was available in the remaining cases. Data on clinical follow-up and radiological assessment were retrieved from the medical record files wherever possible. Thirteen patients suffered from recurrence, in 21 cases, no recurrence was reported upon clinically documented careful follow-up, and in 42 patients, there was insufficient documentation on follow-up.

**Table 1:** Somatic mutations and demographic data

Gene	Average	Sex		Localization		Site			Recurrence	
*n* = 62	Age	Female	Male	Mandible	Maxilla	*R*	*L*	*A*	Yes	No
BRAF (*n* = 34)	42	13	21	33***	1	10	17	7	6	14
Multiple mutations (*n* = 12)	56	3	9	7	5	5	3	4	4*	2
SMO (*n* = 8)	67**	1	7	2	6	6	1	1	0	1
NRAS, HRAS, EGFR (*n* = 3)	48	1	2	2	1	0	3	0	1	0
WT (*n* = 5)	50	2	3	2	3	3	1	1	1	1
Total		20	42	46	16	24	25	13	12	18

*R* right, *L* left, *A* anterior

*p < 0.05 (rho-0.235), **p < 0.01 (rho 0.299), ***p < 0.001

**Table 2:** Somatic mutations and histopathologic features

Gene	Type			Subtype			Secondary subtype			Stroma		Cyst D	Inflm
*n* = 62	Solid	Unicystic	Peripheral	Follicular	Plexiform	Mixed	ACN	BAS	GRAN	*F*	*M*		
BRAF (*n* = 34)	21**	8	5	19***	9	6	7	0	2	27	7	28	11
MULTIGENE (*n* = 12)	12	0	0	7	1	4	6	0	2	8	4	7	2
SMO (*n* = 8)	7	0	1	0	2	6	2	0	0	6	2	7	1
NRAS, HRAS, EGFR (*n* = 3)	2	1	0	0	1	2	0	0	0	3	0	2	1
WILD (*n* = 5)	3	2	0	1	1	2	1	1	0	4	1	5	2
Total	45	11	6	27	14	20	16	1	4	48	14	49	17

*ACN* acanthamatous, *BAS* basaloid, *GRAN* granular, *F* fibroid-desmoplastic-fibrocellular, *M* myxoid-loosen, *Inflm* inflammation

*p ≤ 0.05 (rho 0.216), *p* ≤ 0.01 (rho-0.224)

Radiologically, 46 cases could be assessed. Tumors were evaluated with regard to the following parameters: multi-/unilocularity, relation to the tooth, tooth resorption, border clarity, and cortical expansion. Microscopically, all tissue sections were re-reviewed by S.E.G. and B.S. classified according to the 2017 WHO classification of head and neck tumors into conventional, unicystic, and peripheral types [4] (conventional ameloblastoma was abbreviated as solid in the tables and figures). Further histological subtyping into the three following groups was performed: plexiform > 90% plexiform component present and follicular > 90% follicular component present and mixed when both plexiform and follicular components were present. In addition, acanthomatous, basaloid, and granular tumor changes were documented as well as cystic degeneration with or without inflammatory infiltrate. Stromal alterations such as myxoid and/or fibroid-desmoid were also evaluated.

### Next-generation sequencing

Next-generation sequencing was applied to study 28 different genes: *ARAF*, *BRAF*, *CDK4*, *CDKN2A*, *CTNNB1*, *DDR2*, *EGFR*, *ERBB2*, *FGFR2*, *FGFR3*, *GNA11*, *GNAQ*, *HRAS*, *IDH1*, *KEAP1*, *KIT*, *KNSTRN*, *KRAS*, *MAP2K1*, *MET*, *NFE2L2*, *NRAS*, *OXA1L*, *PDGFRA*, *PIK3CA*, *PTEN*, *RAC1*, and *TP53.* In addition, *SMO* was analyzed by Sanger sequencing (*Supplement*).

All 98 tumor samples were formalin-fixed, decalcified or non-decalcified, and paraffin-embedded according to local practice. Six 10-μm thick sections were cut from FFPE tissue blocks, subsequently deparaffinized, and the tumor areas were macro-dissected from unstained slides using a marked hematoxylin-eosin (H&E)-stained slide as a reference. After proteinase K digestion, DNA was isolated with the Maxwell® 16 FFPE Plus Tissue LEV DNA Purification Kit (Promega, Mannheim, Germany) on the Maxwell® 16 (Promega) following the manufacturer’s instructions. The DNA content was measured using a real-time qPCR-based method.

For multiplex PCR-based target enrichment, the isolated DNA (10 ng each) was amplified with two customized GeneRead DNAseq Targeted Panel V2 (Qiagen, Hilden, Germany) and the GeneRead DNAseq Panel PCR Kit V2 (Qiagen) according to the GeneRead DNASeq Gene Panel Handbook (Qiagen). These two panels comprise a subset of 28 cancer-relevant genes as detailed above.

Libraries were constructed using the Gene Read DNA Library I Core Kit and the Gene Read DNA I Amp Kit (Qiagen). After end-repair and adenylation, NEXTflex DNA Barcodes were ligated (Bio Scientific, Austin, TX, USA). Library products were quantified with Qubit dsDNA HS Assay Kit (Thermo Fisher Scientific, Waltham, MA, USA) on the Qubit 2.0 Fluorometer (Thermo Fisher Scientific), diluted and pooled in equal amounts. Finally, 12 pM of the constructed libraries were sequenced on the MiSeq (Illumina, San Diego, CA, USA) with a MiSeq reagent kit V2 (300-cycles) (Illumina) following the manufacturer’s recommendations.

Data were exported as FASTQ files. Alignment and annotation were done using a modified version of a previously described method [12]. BAM files were visualized in the Integrative Genomics Viewer (http://www.broadinstitute.org/igv/). A 5% cutoff for variant calls was used, and results were only interpreted if the coverage was > 200-fold.

### Sanger sequencing of *SMO*

*SMO* (exon 6) and *SMO* (exon 9) analysis was performed on all 98 tumor blocks using Sanger sequencing. DNA was amplified by PCR with specific primers (exon 6: For 5′- TAACCCACCTTCTGTCCCAC -3′, Rev 5′- TGGCAGCTCCCAGTACTG -3′; exon 9: For 5′- CACCTGTCTACGTTCCCTCA -3′, Rev 5′- GCAGGACCCGACAAAACCTA -3′) and an annealing temperature of 60 °C. PCR products were checked for the expected fragment length of 235 and 296 bp and were purified using Exo I and Fast-AP (Thermo Fisher Scientific, Waltham, MA, USA). Subsequent cycle-sequencing reactions were carried out using the BigDye Terminator v1.1 Cycle Sequencing Kit (Thermo Fisher Scientific) and the 3500 Genetic Analyzer (Thermo Fisher Scientific). PCR and cycle-sequencing were performed twice for each sample. Due to DNA degradation, 14 cases were unavailable for sequencing.

### Statistical analyses

Clinical, radiological, and histological variables were evaluated according to the mutation status. Categorical variables were expressed as a percentage (frequency), and association with mutation status was assessed by using *χ*^2^ (chi-square) test. Non-parametric correlation test (Spearman’s correlation test) was used to analyze the mutation status correlation with clinical, radiological, and histological parameters. Statistical analyses were carried out using the software SSPS.16.0.

## Results

### Histopathological and radiological characteristics

**Fig. 1:**
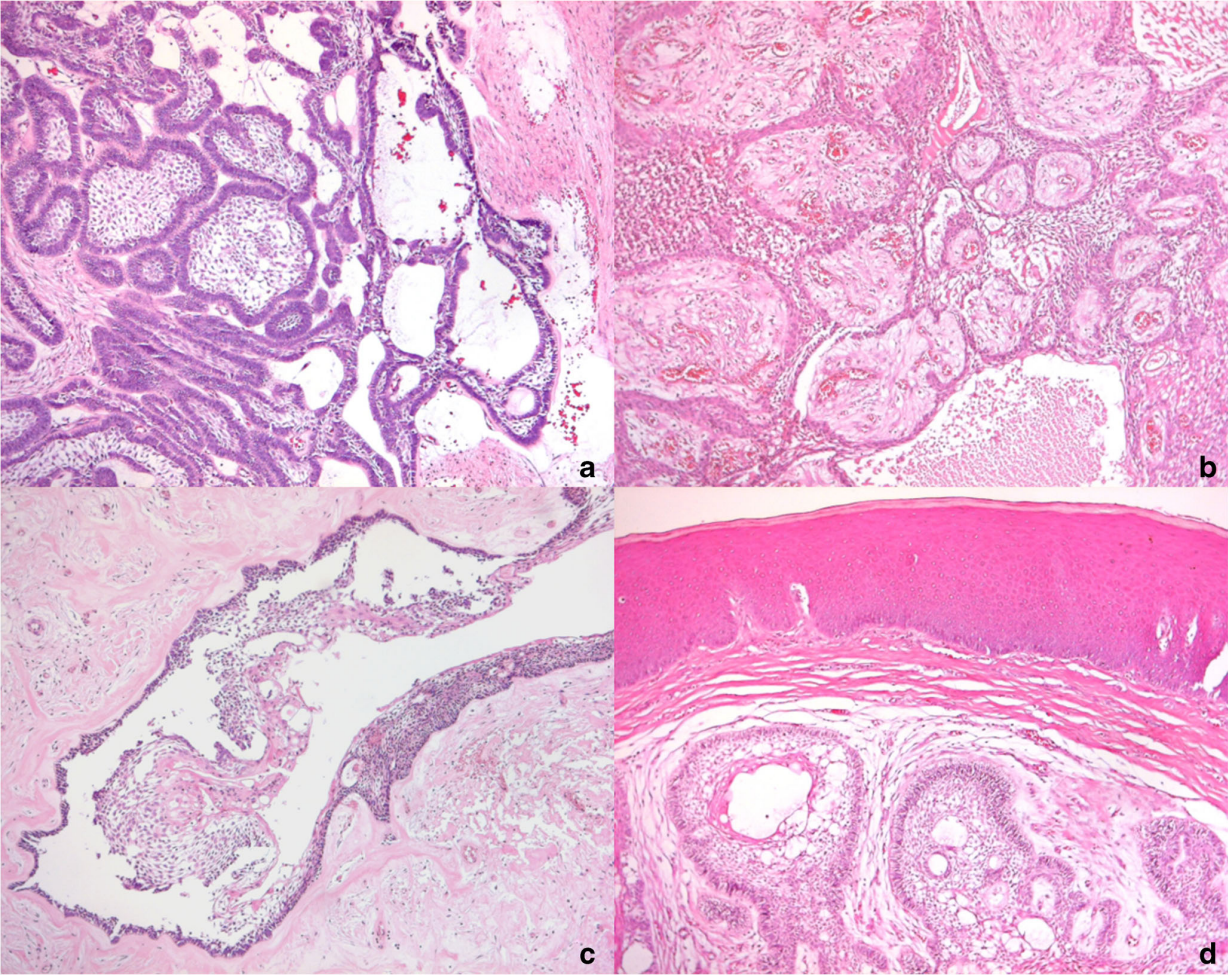
Histologic features of ameloblastomas analyzed in this study. **a** Follicular ameloblastoma showing tumor islands with peripheral columnar cells and stellate reticulum-like cells (H&E, magnification ×100). **b** Plexiform ameloblastoma showing long anatomizing cords of ameloblastic epithelium (H&E, magnification ×100). **c** Intraluminal unicystic ameloblastoma lined by ameloblastic epithelium with luminal projections, no evidence of stromal invasion (H&E, magnification ×100). **d** Peripheral ameloblastoma showing tumor islands just underneath the oral mucosal epithelium (H&E, magnification ×100)

All 76 ameloblastomas were classified according to WHO classification [4] into the following types: 51 conventional, 6 peripheral, and 19 unicystic. Six out of 19 unicystic types were luminal, one intraluminal, and 12 mural (representative histology shown in Fig. 1). Information on recurrences upon careful long-term follow-up (5–15 years) was available from 34 patients (44%). Radiological images and re-examination were possible in 46 cases (Tables 1, 2, and 3).

**Table 3:** Somatic mutations and radiological parameters

Gene	Locularity		Tum Mar		Teeth Rel		Root Res		Cort Exp
	Multi	Uni	Clear	Unclear	Yes	No	Yes	No	
BRAF (*n* = 25)	11**	14	18	7	15	10	9	16	11*
Multigene (*n* = 10)	4	6	6	4	3	7	2	8	9
SMO (*n* = 4)	3	1	4	0	1	3	1	3	0
NRAS, HRAS, EGFR (*n* = 3)	2	1	2	1	1	2	0	3	1
WILD (*n* = 4)	4	0	2	2	2	2	2	2	3
Total	24	22	32	14	22	24	14	32	24

*Tum Mar* tumor margin, *Teeth Rel* teeth relation *Root Res* root resorption, *Cort Exp* cortical expansion

*p ≤ 0.05, **p ≤ 0.01

In 14 cases (18.4%), sequence analysis failed due to massive degradation of DNA. We assume that in these cases, which were received for reference pathology from external laboratories, either non-buffered formalin was used for fixation or acid-based decalcification procedures. Thus, a full data set of NGS analysis in association with clinical, histological, and radiological parameters was available in 62 cases. Eight of 14 cases not available for analysis by NGS (n.a.) were unicystic ameloblastomas mostly of luminal type.

### Frequency of somatic mutations in ameloblastomas

**Fig. 2:**
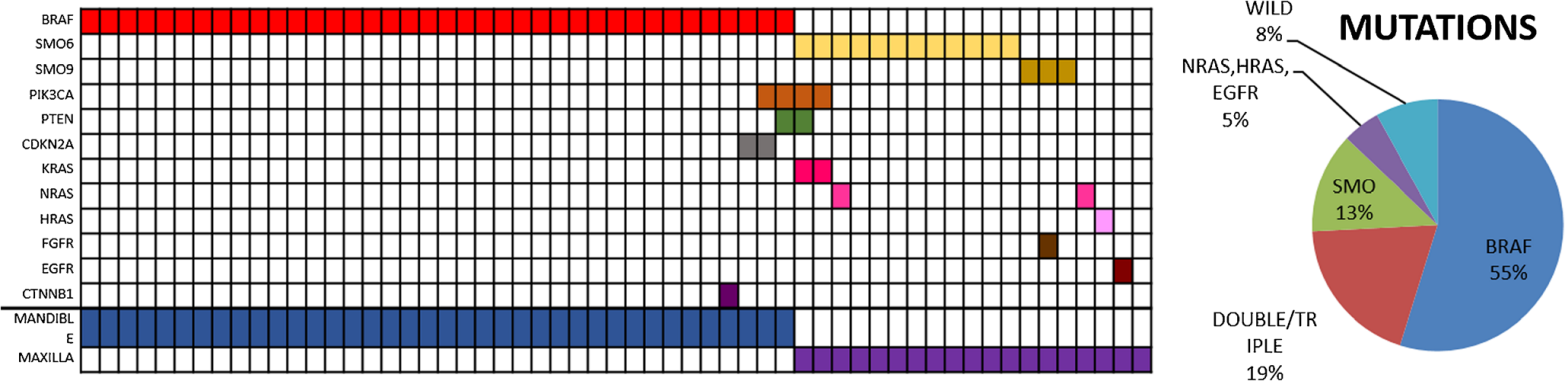
Overview of genomic alterations in ameloblastomas. Distribution of mutated genes with regard to anatomic location. Colored boxes indicate the presence of mutations in the genes listed on the left; columns indicate the respective cases. Prevalence of gene mutations

Mutations were identified in 57 of 62 ameloblastomas (92%) available for comprehensive analysis by NGS. Of these 57 cases, one somatic mutation was observed in 45 cases (79%), while 12 tumors (21%) harbored multiple genetic alterations (two mutations in 11 cases and three mutations in one single case). The *BRAFV600E* mutation was by far the most prevalent alteration detected in 34 of 57 tumors (60%). Mutations in *SMO* were found in 8 of 57 ameloblastomas (14%). Six *SMO* mutations were located in exon 6 and two mutations in exon 9. Single *NRAS*, *HRAS*, and *EGFR* mutations with a wild-type background of *BRAF* and *SMO* were identified in three further cases. However, somatic *KRAS*, *PIK3CA*, *PTEN*, *FGFR*, *CDKN2A*, and *CTNNB1* co-occurred also in the background of either *BRAF*- or *SMO*-mutated ameloblastomas (Fig. 2). In all cases where the COSMIC mutational database did not indicate bona fide somatic oncogene mutations, such as in the case of the *EGFR* mutation, we verified a somatic variant by sequencing normal tissue from the same patient, which failed to detect the mutation.

### Ameloblastomas with mutations in *BRAF*, *SMO* or multiple genes reveal different demographic features

*BRAFV600E*-mutant ameloblastomas presented at a much earlier age (mean age 42 years) as compared to *SMO*-mutated ameloblastomas, which was the oldest patient subgroup (mean age 67 years, rho = 0.299; *p* = 0.019)*.* Male to female ratio was highest in *SMO*-mutant cases (7:1), whereas the lowest ratio was observed in *BRAF*-mutant cases (1.6:1). Strikingly, all ameloblastomas with *BRAF* mutations were exclusively located in the mandible with the exception of one single case (97.1%). We found a clear dichotomy to *SMO*-mutated tumors, which were most frequently located in the maxilla (75%). These differences were statistically highly significant (*p* = 0.000; rho = 0.549).

**Fig. 3:**
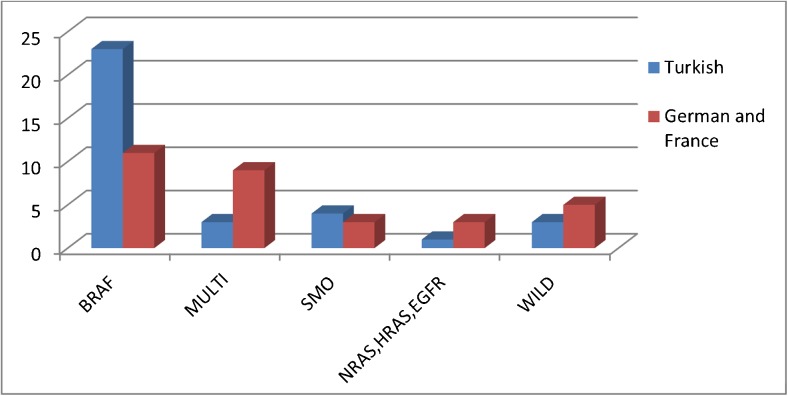
Distribution of mutations with regard to geographic regions. TK cases from Turkey, G + F cases from Germany and France

The distribution of the mutation status revealed remarkable differences with regard to geography (Fig. 3). Ameloblastomas harboring single *BRAFV600E* mutations were significantly more frequent in Turkish (67.6%) than in German and French patients (32.4%). In contrast, mutations in multiple genes were more frequently found in cases from Germany and France (75%), when compared to cases from Turkey (25%). With respect to mutations in *SMO* mutations, there was no geographic difference.

Although reliable clinical data from long-term follow-up was available only in a subset of cases (30 out of 62 cases, 48.4%), there was a clear correlation between the risk of recurrence and the mutational status (rho − 0.235, *p* = 0.033). The recurrence rate was highest in the group of ameloblastomas with double or triple gene mutations. In contrast, tumors with *BRAF* mutations revealed a lower risk for recurrence. For *SMO* mutations, we had too few cases with long-term follow-up to calculate reliable risk of recurrence (Table 1). Thus, our data for the first time suggest that it may be possible to stratify patients for follow-up or for the extent of radical surgical procedures based on their mutational profile.

### Genotype-phenotype correlation

Table 2 summarizes the histological parameters of all mutated and wild-type ameloblastomas. The histologic classification of the tumors comprehended conventional (*n* = 45), unicystic (*n* = 11), and peripheral (*n* = 6) cases. One of 11 unicystic ameloblastomas was of luminal type. Tumors with *BRAF* mutations were found in all three histological groups, whereas neither *SMO* nor multigene mutations were seen in unicystic type. Moreover, multiple gene mutations were exclusively present in solid ameloblastomas (100%). Peripheral type ameloblastomas revealed exclusively single somatic mutations in *BRAF* or *SMO* (rho − 0.224, *p* = 0.010).

Evaluation of histological subtypes showed that the majority of the cases were follicular (*n* = 27), followed by mixed (*n* = 20) and plexiform (*n* = 14) subtypes. Most follicular subtypes showed *BRAF* or multiple gene mutations, whereas most plexiform and mixed variants harbored single mutations in *SMO*, *NRAS*, *HRAS*, or *EGFR*. This difference was statistically significant (*p* = 0.006). Spearman correlation test showed significant positive correlation between mutation status and histologic subtype (rho 0.216, *p* = 0.05, Fig. 1a–e). Of the 62 cases, cystic degeneration was seen in 49 cases, either in the tumor islands or as a real cyst form. Inflammation was present in 1 out of 62 cases.

### Radiological parameters and mutational status

Radiological re-evaluation was performed in 46 of 62 NGS performed ameloblastomas (Table 3). The locularity pattern of these lesions was almost equally distributed (24 multi- vs 22 unilocular cases), whereas tumor margins were clearly demarcated in the majority of ameloblastomas (69.5%). Almost half of the tumors showed relation with the teeth (47.8%) and root resorption was observed in 14 out of 46 cases (30.4%).

Remarkably, the unilocular to multilocular pattern rate was higher (1.3:1, 1.5:1, respectively) in the tumors with *BRAF* and multiple gene mutations, in contrast to *SMO*, *NRAS*, *HRAS*, and *EGFR*-mutated ameloblastomas (1:3, 1:2, respectively). Moreover, the wild-type ameloblastomas revealed always a multilocular radiological pattern (100%), (*p* = 0.007). The tumor margins were clearly demarcated in all *SMO*-mutated cases (in good agreement with the absence of recurrence in these cases), whereas the highest rate of unclear tumor margins were observed in wild-type ameloblastomas. Cortical expansion was prominent in ameloblastomas with *BRAF* or multiple gene mutations, but *SMO*-mutated tumors never revealed cortical expansion (*p* = 0.028).

## Discussion

Our data confirm previous studies who found that *BRAF* and *SMO* are by far the most frequent oncogenic driver mutations in ameloblastomas. These genetic alterations lead to constitutive activation of MAP kinase and hedgehog signaling pathways, respectively [6–8].

We also confirm highly significant phenotypic differences in these two types of ameloblastomas as *BRAF*-mutant cases occurred preferentially in the mandible and at a much younger age (mean age 42 years) than *SMO*-mutant cases occurring preferentially in the maxilla at an older age (mean age 67 years). This data underscores an emerging appreciation of the anatomical specificity of driver mutations, which reflect distinctive odontogenic pathways in the upper and lower dentition [13]. There is evidence that the nature of the molecular signaling in the upper and lower jaws may vary. The dental formula is the same in both arches in mice and in humans, but the shape and morphologies of the homologous teeth in the two jaws are clearly distinct. Biochemical signaling differences have been demonstrated in the mouse for Dlx-1 and -22 [14] and also for the activin/follistatin genes [15]. Although it is not known how neural crest-derived cells migrating into the developing maxillary and mandibular regions develop the ability to respond differently to ectodermal signaling, reports of apparently independent genetic determination of maxillary and mandibular dentitions, based on tooth size data derived from twins, are consistent with the molecular evidence [16]. Assuming that mutations in transcriptionally active genes controlling expansion of ameloblasts occur in a stochastic manner, the genetic landscape of ameloblastomas provides additional evidence for the existence of distinct developmental cues during ameloblast expansion. In this context, it is interesting to note that we found multiple gene mutations only in European but not in Turkish patients. As there are no known environmental factors predisposing to ameloblastomas, it remains to be speculated that there are differences in genetic backgrounds defining different mutational spectra.

Interestingly, the hedgehog signaling pathway is also involved in pituitary formation during early vertebrate embryogenesis. Its activation is triggered by hedgehog ligand binding to a receptor complex formed by the transmembrane protein patched 1 (PTCH1). In the presence of the ligand, the frizzled class receptor, smoothened (SMO), is released from PTCH1 inhibition and activates the transcription factor gene family GLI1, GLI2, and GLI3 [17]. After the appearance of Rathke’s pouch, Sonic HH (SHH) expression is excluded from this region but remains in surrounding areas [16]. As different types of mutations in the hedgehog, the Wnt, and BRAF/MAPkinase pathways define distinct subtypes of craniopharyngiomas [18], there seems to be signaling analogy in the pathways driving dentition and Rathke’s pouch formation [19]. Craniopharyngiomas are generally considered to arise from the remnants of Rathke’s pouch or a misplaced enamel organ [19]. Gomes et al. [18] hypothesized that crosstalk between Wnt/β-catenin and SHH pathways, which are important during pituitary embryogenesis, could contribute to the imbalance in intracellular signaling in the molecular pathogenesis of adamantinomatous craniopharyngiomas. Taken all together, we may speculate that ameloblastomas and craniopharyngiomas share similar tumorigenic pathways and SMO-mutated ameloblastomas may resemble a distinct subtype of craniopharyngiomas with special reference to maxillary location.

In line with these molecular subtypes, we found a clear correlation between the risk of recurrence and the mutational status. The recurrence rate was highest in the group of ameloblastomas with multiple gene mutations. Tumors with *BRAF* mutations revealed significantly lower risk for recurrence, and tumors with *SMO* gene mutation appear to be associated with higher recurrence [8]. Thus, our data suggest that a stratified clinical management of ameloblastomas may be possible. Tumors with *BRAF* mutations are excellent candidates for neoadjuvant BRAF inhibitor treatment followed by limited surgical treatment in tumors with single *BRAF* mutations and extensive resection of tumors with *BRAF* and concurrent multiple mutations. In contrast *SMO* mutant, tumors require a priori definite surgical resection with wider margins as they carry a high risk of recurrence.

Our data also raise the possibility that there is a continuum from benign to locally recurrent ameloblastomas to ameloblastic carcinomas. While malignant ameloblastoma showing clear features of malignancy is generally accepted to be a different entity based on its ability to metastasize, it is possible that the histologically benign but “metastasizing ameloblastoma” might be one of the lesions accumulating several oncogenic mutations and thus, acquiring a higher potential for malignant growth. Therefore, a larger number of biologically malignant ameloblastomas need to be analyzed by deep sequencing in order to establish such a relationship.

Finally, we here describe a genotype-phenotype correlation as tumors with *BRAF* mutations were found in all three histological groups, whereas neither *SMO* nor multiple gene mutations were seen in unicystic type. Moreover, multiple gene mutations were exclusively present in solid ameloblastomas (100%). Peripheral type ameloblastomas revealed single somatic mutations in *BRAF* or *SMO*. Most follicular subtypes showed *BRAF* and multiple gene mutations, whereas most plexiform and mixed variants harbored either *SMO*, *NRAS*, *HRAS*, or *EGFR* mutations. Therefore, our results support the argument that the mutation status may be related to the histological pattern (follicular versus plexiform). In their study, Sweeney et al. [8] found that plexiform variants had a *SMO* mutation (*p* < 0.02), while most follicular and desmoplastic variants carried either *SMO* or *BRAF* mutation.

In summary, our data significantly extend previous studies and provide evidence that there are distinct molecular pathways driving ameloblastomas with different histological and clinical features possibly requiring different approaches for clinical management.
